# Core promoter acetylation is not required for high transcription from the phosphoenolpyruvate carboxylase promoter in maize

**DOI:** 10.1186/1756-8935-2-17

**Published:** 2009-12-02

**Authors:** Ina Horst, Sascha Offermann, Bjoern Dreesen, Markus Niessen, Christoph Peterhansel

**Affiliations:** 1 Leibniz University Hannover, Institute of Botany, 30419 Hannover, Germany; 2 Washington State University, Pullman, Washington, USA; 3 RWTH Aachen, Biology I, 52056 Aachen, Germany

## Abstract

**Background:**

Acetylation of promoter nucleosomes is tightly correlated and mechanistically linked to gene activity. However, transcription is not necessary for promoter acetylation. It seems, therefore, that external and endogenous stimuli control histone acetylation and by this contribute to gene regulation. Photosynthetic genes in plants are excellent models with which to study the connection between stimuli and chromatin modifications because these genes are strongly expressed and regulated by multiple stimuli that are easily manipulated. We have previously shown that acetylation of specific histone lysine residues on the photosynthetic phosphoenolpyruvate carboxylase (*Pepc*) promoter in maize is controlled by light and is independent of other stimuli or gene activity. Acetylation of upstream promoter regions responds to a set of other stimuli which include the nutrient availability of the plant. Here, we have extended these studies by analysing histone acetylation during the diurnal and circadian rhythm of the plant.

**Results:**

We show that histone acetylation of individual lysine residues is removed from the core promoter before the end of the illumination period which is an indication that light is not the only factor influencing core promoter acetylation. Deacetylation is accompanied by a decrease in gene activity. Pharmacological inhibition of histone deacetylation is not sufficient to prevent transcriptional repression, indicating that deacetylation is not controlling diurnal gene regulation. Variation of the *Pepc *promoter activity during the day is controlled by the circadian oscillator as it is maintained under constant illumination for at least 3 days. During this period, light-induced changes in histone acetylation are completely removed from the core promoter, although the light stimulus is continuously applied. However, acetylation of most sites on upstream promoter elements follows the circadian rhythm.

**Conclusion:**

Our results suggest a central role of upstream promoter acetylation in the quantitative regulation of gene expression in this model gene. Induced core promoter acetylation is dispensable for the highest gene expression in the diurnal and circadian rhythm.

## Background

Acetylation of lysines on the N-terminal tails of histones shows a very high degree of correlation with gene transcription in genome-wide analyses of microbes and mammals [1]. Although comprehensive data for individual acetylation sites are not available from plants, many studies of individual genes, or groups of genes, indicate that this correlation is also conserved in the green lineage. Studies of gene induction by light in *Arabidopsis *mutants with defects in histone acetyltransferases revealed a requirement of histone acetylation for light-activated gene transcription [2]. Guo *et al*. suggested that H3K9 acetylation in *Arabidopsis *is required for the binding of RNA Polymerase II to promoters of light-regulated genes [3] and a similar scenario has been suggested for H3K14 acetylation and transcription on the seed-specific Opaque2 gene in maize [4]. A tight correlation of H4 acetylation and gene activity was also observed in a comparative study of more than 50 tobacco genes [5].

Two models have been suggested for the function of histone acetylation in gene expression: On the one hand, acetylation neutralizes the positive charge of lysine side chains and, by this, reduces the electrostatic interactions with the negatively charged DNA backbone (charge neutralization model, [6]). On the other hand, acetylated histones provide binding sites for bromodomain proteins such as chromatin remodelling complexes and general transcription factors [7]. Thus, the pattern of acetylation, together with other histone modifications, might provide a histone code that is read out by other proteins that consequently control transcription [8]. The code can store and integrate information about environmental and endogenous stimuli that are important for the regulation of gene activity.

The phosphoenolpyruvate carboxylase (*Pepc*) gene in maize is an excellent model for the analysis of signal integration on promoters because it is expressed at very high levels and is strongly regulated on the transcriptional level by multiple stimuli. The gene is exclusively active in the mesophyll cells of leaves but inactive in the directly adjacent bundle sheath cells or in other tissues, such as roots [9]. Furthermore, transcription is activated by light and modulated by the availability of nutrients and the metabolic state of the cell [10,11]. We have deciphered the function of histone modifications in the transcriptional regulation of this gene [12-14]. Before the first illumination, the *Pepc *promoter shows low basal activity in leaves - most promoter acetylation sites are already acetylated at this stage. Light specifically induces acetylation of H4K5 and H3K9 on the core promoter. This is accompanied by the induction of transcription, although transcription is not necessary for acetylation. The situation is complicated under conditions where metabolic stimuli act on the promoter. Low nitrogen levels or high leaf sugar contents are sufficient to efficiently suppress promoter activity. However, histone modifications on the core promoter region, which is proximal to the transcription initiation site and which contains all binding sites for the basal transcription machinery, do not respond to these stimuli. Instead, metabolic stimuli control the acetylation of a distal promoter element that is more than 1000 bp upstream of the transcription initiation site. Here, all tested acetylation sites are affected to a similar degree and a good quantitative correlation between the upstream promoter acetylation and the transcription rates can be observed. Again, acetylation levels are not a consequence of transcription levels, but can also be modulated when transcription is inhibited by pharmacological treatments. Interestingly, histone methylations are not affected by any of these stimuli. They are exclusively controlled by a tissue-specific factor that induces high trimethylation of H3K4 in leaf mesophyll cells. Moreover, H3K4 methylation is restricted to the core promoter and is absent from the upstream promoter [12].

In this study, we used diurnal and circadian promoter controls to further unravel the interrelationship of promoter histone acetylation and transcription in this gene. Our results question the mechanistic connection between histone acetylation and transcription, in spite of their tight correlation under standard conditions.

## Results

### Diurnal promoter activity and histone acetylation on *Pepc*

**Figure 1:**
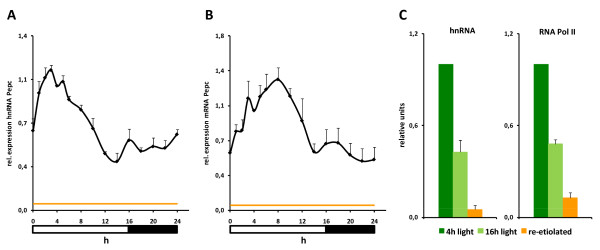
**Diurnal pattern of phosphoenolpyruvate carboxylase (*Pepc*)**. Quantification of (A) *Pepc *hnRNA and (B) *Pepc *mRNA through one day/night cycle. Numbers are units relative to levels in leaves harvested 4 h after illumination. The orange line shows the transcription level in re-etiolated leaves (lowest *Pepc *promoter activity) for comparison. (C) Comparison of hnRNA levels and RNA Polymerase II abundance in the *Pepc *coding region. Data points are based on at least three independent experiments. Vertical lines indicate standard errors.

**Figure 2:**
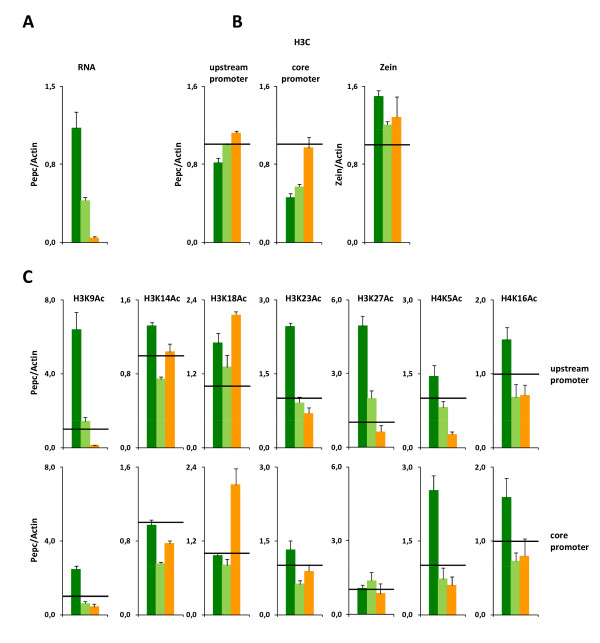
**Diurnal histone acetylation pattern on the upstream and core phosphoenolpyruvate carboxylase (*Pepc*) promoter**. (A) Transcription levels, (B) nucleosome density and (C) histone acetylation in leaves 4 h after illumination (dark green columns), 16 h after illumination (light green columns), or re-etiolated leaves (orange columns), respectively. Values are H3K9, H3K14, H3K18, H3K23, H3K27, H4K5 and H4K16 histone acetylation levels on an upstream (-1300 bp) and a core (-200 bp) promoter position of *Pepc*. Data are standardized for acetylation levels on the *Actin*-1 promoter. For better orientation, the 1.0 level is emphasized by a black line. H3C = chromatin precipitated with an antibody to an invariant epitope on histone H3 as an indicator for nucleosome density. *Zein *= Intergenic region within the *Zein *gene cluster. Data points are based on four independent experiments. Vertical lines indicate standard errors.

The maize *Pepc *gene is highly active in the leaves of illuminated plants. The activity is only slightly reduced during a normal dark period [13]. The diurnal pattern of gene expression was unknown. We therefore studied the promoter activity (amount of unspliced heterogenous nuclear transcripts [hnRNA]) and mRNA accumulation during the day (Figure 1). Promoter activity was induced two- to threefold by illumination and reached its highest levels around 4 hours after illumination (hai). Afterwards, a constant decline down to a basal level was observed that remained almost constant during the night. mRNA accumulation followed a similar profile with an offset of approximately 4 h (Figure 1B). It should be noted that the lowest levels of *Pepc *promoter activity obtained in the natural diurnal rhythm are still at least as high as the activity of the constitutively expressed *Actin*-1 gene [15] and much higher than lowest *Pepc *promoter activities observed in re-etiolated leaves or roots (orange line, and such-like [14]). We verified that heterogeneous nuclear RNA (hnRNA) measurements are representative of the transcription rate by comparing with the number of RNA polymerase II molecules on the coding region of the gene as determined by chromatin immunoprecipitation (Figure 1C). Results obtained by both methods were very similar, indicating that the hnRNA method is suitable for the detection of promoter activity. The data indicate that *Pepc *promoter activity varies around a high level during the day. We expected this variation to be accompanied by changes in histone acetylation and so, accordingly, tested the acetylation levels on the upstream and core promoter regions. Data for all sites of H3 N-terminal acetylation (H3K9, H3K14, H3K18, H3K23 and H3K27) and for the acetylation of lysine 5 and 16 on histone H4 at two typical positions of the upstream and core promoter, respectively, are shown in Figure 2. Additionally, detailed promoter acetylation profiles for each modification are available in the Additional File 1. An antibody to an invariant domain of histone H3 (H3C) was used to estimate the nucleosome densities on the gene [1]. Additional File 2 shows that profiles obtained with this antibody are very near to those obtained with a second independent antibody directed to an invariant domain of H4. Data were recorded at 4 hai (the highest diurnal promoter activity) and 16 hai (the lowest diurnal promoter activity). For comparison, data from re-etiolated plants are shown. We refer to differences between 4 hai and the re-etiolated state as a response to prolonged darkening and to differences between 4 hai and 16 hai as a diurnal response. The black line always indicates the levels obtained on the constitutively active *Actin*-1 reference gene.

As summarized in Figure 2A, the response of transcription to prolonged darkening was much stronger than the diurnal response. Nucleosome densities were almost constant on the upstream promoter and close to those observed on *Actin*-1 (Figure 2B). On the core promoter, the differences in nucleosome densities were obvious. In the re-etiolated state, where the gene shows minimal activity, nucleosome density was similar to *Actin*-1. A reduction of around 40% relative to this value was observed at the basal or induced activation, state. As a control, we also measured nucleosome densities on the *Zein *gene, which is exclusively expressed in developing seeds but never in leaves. Here, nucleosome densities are around 30% higher than on *Actin*-1. These data suggest that the *Pepc *promoter is always in an open chromatin configuration in leaves. Nucleosome density on the core promoter is even further reduced when the gene is fully activated.

Highly individual patterns, depending on the promoter position, were observed for the tested acetylation sites (Figure 2C). On the upstream promoter region, a good correlation of acetylation and transcription was evident for only three of the modifications (H3K9ac, H3K27ac, H4K5ac). For other modifications (H3K14ac, H3K18ac, H3K23ac, H4K16ac), the diurnal response was as strong, or even stronger, than the response to prolonged darkening. On the core promoter, more modification sites showed only weak or no responses to the stimuli applied. Where a light response was evident (H3K9ac, H3K23ac, H4K5ac, H4K16ac), the diurnal response was as strong as the response to prolonged darkening. Thus, the lowest acetylation levels for these sites were already observed at 16 hai, where significant gene transcription occurred. Remarkably, the lowest acetylation levels on *Pepc *are still in the range of those observed on the constitutively active *Actin*-1 gene. The data reveal a complex diurnal control of acetylation on both promoter regions. Only some of the acetylation sites on the upstream promoter showed a positive correlation of acetylation and transcription. Acetylation of all variable sites on the core promoter is reduced to minimum levels in the diurnal rhythm, although transcription remains high. Nucleosome density appears to respond mainly to stronger promoter inactivation down to the lowest levels observed in leaves. This is reminiscent of the light-induced enhancement in chromatin accessibility to the restriction enzymes that we observed in an earlier study [16].

### Pharmacological hyperacetylation and diurnal regulation of *Pepc*

**Figure 3:**
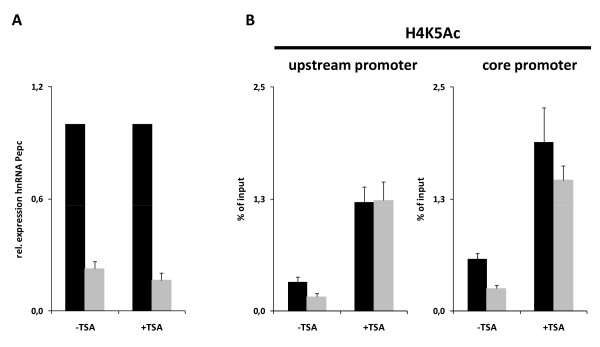
**Diurnal transcription after HDAC inhibition**. (A) Quantification of phosphoenolpyruvate carboxylase (*Pepc*) heterogeneous nuclear RNA after treatment with the histone deacetylase inhibitor Trichostatin A. Numbers are values relative to leaves from plants 4 h after illumination. (B) H4K5 acetylation at an upstream (-1300 bp) and a core (-200 bp) promoter position of *Pepc*. Black columns = leaves from plants 4 h after illumination; light grey columns = leaves from plants 16 h after illumination. Data points are based on four independent experiments. Vertical lines indicate standard errors.

In order to test whether the decrease in histone acetylation during the illumination period controls the concomitant modest reduction in promoter activity, detached leaves were treated with the deacetylase inhibitor Trichostatin A (TSA). Figure 3B exemplarily shows the effect of this treatment on H4K5 acetylation. As expected, high levels of histone acetylation were induced in both promoter regions. The reduction in acetylation in the afternoon was almost completely eliminated. Identical results were obtained for all other acetylation sites (data not shown). However, the reduction in promoter activity between 4 hai and 16 hai was completely unaffected by this treatment (Figure 3A). This indicates that high histone acetylation is not sufficient to clamp the promoter on the highest activity levels.

### Circadian promoter activity and histone acetylation on *Pepc*

**Figure 4:**
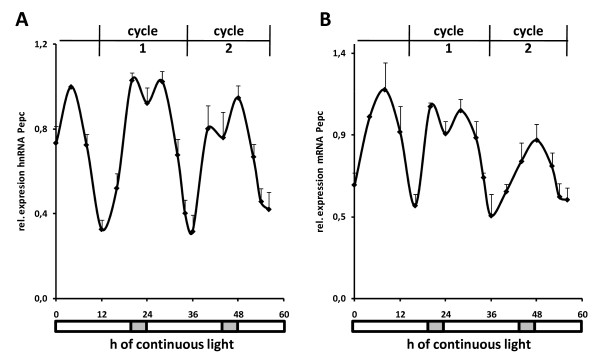
**Circadian transcription profile of phosphoenolpyruvate carboxylase (*Pepc*)**. Quantification of (A) *Pepc *hnRNA and (B) *Pepc *mRNA during 56 hours of constant illumination. After a normal 16 h light period, illumination was extended for an additional 40 h without any dark period or temperature shift. Dark periods during previous growth are symbolized by the light grey bar under each chart. Numbers are values relative to transcription levels in leaves harvested 4 hours after illumination. Data points are based on four independent experiments. Vertical lines indicate standard errors.

Diurnal gene regulation is often affected by circadian oscillators that control the variation in gene transcription during the course of a day, independent of a day/night shift [17]. In plants, circadian control is typically tested by following the promoter activity through a prolonged period of constant illumination. We recorded *Pepc *promoter activity and mRNA accumulation through two daily cycles where dark periods were replaced by constant light (Figure 4). Throughout the experiment, oscillation of promoter activity could be clearly observed. The period of one cycle declined from 24 h to approximately 21 h during the constant light period. This is typical for circadian control of gene transcription [18]. Therefore, diurnal cycling of *Pepc *transcription showed all characteristics of circadian control.

**Figure 5:**
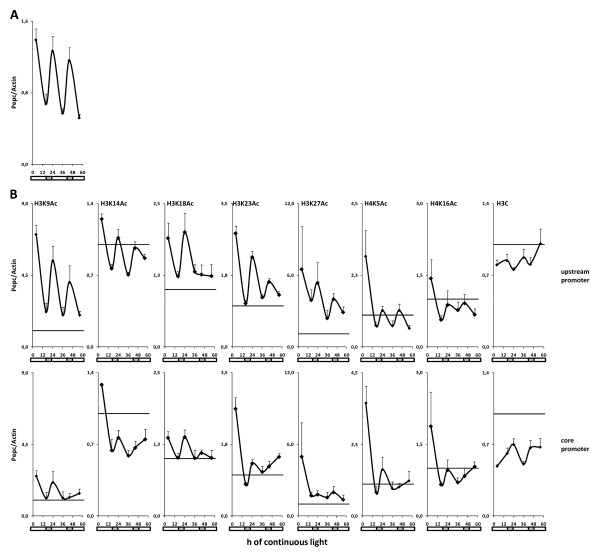
**Circadian histone acetylation profile of the upstream and core phosphoenolpyruvate carboxylase (*Pepc*) promoter**. (A) Transcription levels and (B) histone acetylation under constant illumination (free-running conditions). After a normal 16 h light period, illumination was extended for an additional 40 h without any dark period or temperature shift. Dark periods during previous growth are symbolized by the light grey bar under each chart. The amount of *Pepc *heterogeneous nuclear RNA was standardized for the amount of *Actin*-1 mRNA (A). The amount of promoter chromatin precipitated with the antibody indicated in the figure was standardized for the amount of *Actin*-1 promoter chromatin precipitated with the same antibody. For a better orientation, the 1.0 level is emphasized by a black line. Data for an upstream (-1300 bp) and a core promoter (-200 bp) position are shown. Data points are based on four independent experiments. Vertical lines indicate standard errors.

Histone acetylation was recorded at six indicative time points during constant illumination. Data are shown for a typical position on the core and the upstream promoter, respectively (Figure 5). Results for seven additional promoter positions are displayed in Additional file 3. On the upstream promoter, all tested sites showed circadian oscillation of acetylation, albeit to different extents. The best correlation to transcription, with respect to amplitude and period of oscillation, was observed for H3K9ac. H3K18ac constituted an exception as oscillation was completely suppressed in the second cycle. Very weak oscillation was observed for the invariant control H3C position. On the core promoter, less circadian oscillation of acetylation was clearly observed. Most modifications already showed a clear decline in histone acetylation in the first cycle and no oscillation of acetylation was detectable during the second cycle. As observed in the diurnal rhythm, the lowest acetylation levels on *Pepc *were in the range of, or slightly lower than, those observed on the *Actin*-1 promoter used as a standard. Importantly, the highest transcription levels at the beginning of the second cycle were induced although core promoter acetylation levels remained unchanged. This indicates that the induction of the core promoter acetylation is not mechanistically required for high promoter activity.

## Discussion

### Induced transcription from a deacetylated core promoter

We have previously shown that several histone acetylation sites on the core promoter of the *Pepc *gene in maize are constitutively acetylated in leaves but not in roots or other organs where the gene is always inactive [14]. Light specifically induces acetylation of H3K9, H4K5 [14], H3K23 and H4K16 (this study). Light-induced acetylation of H4K16 is in disagreement with our previous data. However, in earlier studies, leaves from plants 8 hai were investigated. *Pepc *transcription has already decreased at this time point (Figure 1) and, therefore, we might have missed the time point of maximum acetylation in previous experiments.

Our analysis of core promoter acetylation in the diurnal and circadian rhythm revealed that light-inducible acetylation is not required for high-level transcription. In the diurnal rhythm, all light-inducible acetylation sites were deacetylated during the second half of the day. Only the leaf-specific acetylation of H3K14, H3K18 and H3K27 remained stable under these conditions (Figure 2). Gene transcription also declined in the afternoon but remained at an intermediate level that was much higher than the lowest transcription levels found in re-etiolated leaves (Figure 1). Thus, light-inducible acetylation was actively removed from the core promoter during the light period with little impact on transcription. The uncoupling of transcription and acetylation was even more evident under free-running conditions (constant illumination). Particularly at the beginning of the second daily cycle, acetylation of all tested sites on the core promoter remained low but transcription was induced (Figure 5). This indicates that the *Pepc *gene can be activated to maximum transcription levels at low core promoter acetylation levels. The results are particularly significant because *Pepc *is not a weakly expressed gene, but belongs to the highest expressed genes in plants [9]. However, when compared to the constitutively active *Actin*-1 gene, the promoter acetylation at the lowest activation state of *Pepc *was still in the same range as *Actin*-1 (Figures 2C and 5). A similar pattern was observed for nucleosome densities (Figure 2B). This indicates that *Pepc *retains an open promoter chromatin structure even at minimal transcription levels. Thus, modulation of *Pepc *transcription in the circadian rhythm takes place in a euchromatic chromatin context, even if acetylation levels are extremely low with respect to *Pepc*.

Histone acetylation has been correlated, as well as mechanistically linked to transcription (see Background). However, in the circadian rhythm we observed the highest *Pepc *transcription levels at the lowest *Pepc *acetylation. At least two mechanisms for gene activation independent of histone acetylation have been described. Kato *et al. *have shown that the activity of histone chaperones can stimulate promoters which are, to a certain extent, independent of histone acetylation [19]. For enhancer elements, acetylation was dispensable for the activation of associated genes if the underlying DNA sequence contained a sufficiently strong enhancer element [20]. In such cases, remodelling complexes and transcription factors were directly recruited that would normally interact with acetylated histones via their bromodomain [21]. Both described mechanisms might help in the activation of the *Pepc *promoter in maize, particularly as transcription is not activated from the minimum level, but from an intermediate state (Figures 1 and 4). The data underscore our previous observations that specific stimuli induce individual histone modifications on the *Pepc *core promoter whether or not transcription is finally induced. Such a pattern is consistent with a *bona fide *histone code [8]. However, the regulation of acetylation in this promoter region seems to be more complex than anticipated, because diurnal and circadian factors impact on light-induced acetylation.

### Upstream and core promoter regions respond differently to diurnal and circadian stimuli

Acetylation of the tested lysine residues on the upstream promoter differed considerably from core promoter acetylation as it correlated well with transcription levels. This was almost always independent of the stimulus regulating the promoter. The only clear exception to this rule was light regulation of the upstream promoter acetylation. If the promoter was deactivated by extended dark periods, H3K14 and H3K18 acetylation remained high (Figure 2). The general correlation of the upstream promoter acetylation and transcription was also reproducible in diurnal and, mostly, in circadian regulation (Figures 2 and 5). Thus, a charge neutralization model (see Background) can describe most of the interdependence of upstream promoter acetylation and transcription. In such a simple scenario, artificial hyperacetylation should be sufficient to prevent the afternoon decrease in *Pepc *transcription during the diurnal cycle. However, TSA treatments did not affect the diurnal transcription rhythm, albeit inducing strong acetylation of all lysine residues investigated in this study (Figure 3 and data not shown). Both in mammals and plants, positive and negative examples exist for the regulation of gene transcription by TSA. MHC class II genes [22] or the PDK4 gene [23] in mammals can be induced by TSA treatments. In *Arabidopsis*, only few genes were affected by TSA when transcriptome-wide studies of either leaves or germinating seedlings were performed [24,25]. This might indicate that the pleiotropic effects of this treatment were masking gene-specific induction or that the acetylation level itself did not contain the relevant information for gene regulation. Consistent with the latter model, Clayton *et al*. suggested that high turn-over rates, rather than steady-state acetylation levels, are important for high transcription levels [26]. We conclude that, for the *Pepc *promoter, diurnal fluctuations in transcription are not controlled by the level of upstream promoter acetylation.

There is significant evidence from other systems that the diurnal and circadian regulation of transcription is controlled by changes in histone acetylation. In plants, the promoter of the clock component TOC1 is regulated by acetylation/deacetylation cycles that are controlled by the endogenous clock itself [18,27]. In mammals, the central clock component CLOCK is a histone acetyltranferase that binds in a multiprotein complex to promoters containing E-box DNA elements [28]. Interestingly, a G-box (the plant functional homologue to the mammalian E-box) is present in the upstream promoter at position -1186, but not in the core promoter of *Pepc*. It is therefore plausible to assume that upstream promoter elements are more important for the quantitative modulation of *Pepc *transcription levels than the core promoter. Rather, the latter might integrate information on basal factors such as illumination status and tissue specificity. This is in agreement with the observation that *Pepc *core promoter sequences were sufficient to control leaf-specific and light-dependent transcription when linked to reporter genes [29,30].

## Conclusion

In conclusion, the analysis of histone modifications and promoter activity under specific growth conditions allowed for the uncoupling of core promoter acetylation and transcription on a model gene in maize. Individual acetylation sites are differentially regulated dependent on the stimulus and the promoter position.

## Methods

### Sequences

The *Pepc *sequence was derived from GenBank accession gi 22396. BAC clone gi 116268332 was used to add 5' sequence elements. The *Actin*-1 promoter was described in [15]. The *Zein *gene sequence was derived from Genbank accession gi 535019 [16].

### Plant material and growth condition

Maize (*Zea mays *cv. Montello) was cultivated in growth chambers with a 16-h photoperiod and a day/night temperature regime of 25°/20°C. The plants were illuminated with Osram Superstar HQI-T 400W/DH lamps at a photon flux density of 120-180 μmol m^-2 ^s^-1^. Seedlings were grown in soil (VM, Einheitserde, Sinntal-Jossa, Germany) for 10-12 days. Re-etiolated plants were derived from illuminated plants that were subjected to darkness for 72 h. For constant light experiments, the dark period was replaced by continuous illumination with constant temperature.

### Trichostatin A treatment

After 1 h of illumination, 10-12-day-old leaves were detached under water 1 cm above the laminar joint and incubated for 3 h (4 hai) or 15 h (16 hai) in tap water containing 30 μM trichostatin A (TSA; International Clinical Services, Munich, Germany). To avoid nitrogen depletion, 0.275 μM trans-Zeatin (Sigma-Aldrich, Schnellendorf, Germany) and 16 mM KNO_3 _were added [14,31]. Mock control leaves were incubated in tap water with Zeatin, KNO_3 _and methanol.

### Crosslinking and chromatin immunoprecipitation (ChIP)

As described previously, 6 g of foliar leaf material were harvested and crosslinked [13,14]. ChIP was performed as described by Bowler *et al. *[32] with modifications as described in Haring *et al*. [15]. Chromatin was sheared with a Bioruptor (Diagenode) for 10 min (setting: high, interval 30/30 s) under constant cooling. Modified histones were detected with 5 μl anti-acetyl H4K5 (Upstate 07-327 Millipore, Schwalbach, Germany), 5 μl anti-acetyl H4K16 (Upstate 07-329), 5 μl anti-acetyl H3K9 (Upstate 07-352), 1 μl anti-acetyl H3K14 (Upstate 07-353), 1 μl anti-acetyl H3K18 (Upstate 07-354), 5 μl anti-acetyl H3K23 (Upstate 07-355), 5 μl anti-acetyl H3K27 (Upstate, 07-360),1 μl anti H3 C-term (Abcam ab1791) and 7.5 μl H4 C-term (Abcam 10158). RNA polymerease II was detected with 7.5 μl of an antibody directed to the C-terminal domain repeat (Abcam 817). The control serum for determination of background precipitation was derived from rabbits immunized with an unrelated protein from potato. In general, background signals never exceeded 10% of positive signals.

### RNA preparation and reverse transcription

RNA isolation and reverse transcription were performed as described [13]. As a direct estimate for promoter activity, hnRNAs were amplified from cDNA with a primer system specific for an intron [33,34]. A dilution series of illuminated leaf cDNA was used as a standard. Oligonucleotide sequences and conditions are given in Additional File 4.

### Quantitative real-time polymerase chain reaction (PCR)

Quantitative PCR was performed on an ABI PRISM 7300 (Applied Biosystems) using SYBR Green fluorescence (Platinum SYBR Green qPCR Mix, Invitrogen) for detection. Oligonucleotide sequences and conditions are given in Additional File 4.

## Abbreviations

ChIP: chromatin immunoprecipitation; hai: hours after illumination; hnRNA: heterogeneous nuclear RNA; *Pepc*: phosphoenolpyruvate; PCR: polymerase chain reaction; TSA: Trichostatin A.

## Competing interests

The authors declare that they have no competing interests.

## Authors' contributions

IH, SO, BD and CP conceived of and designed the experiments. IH, SO, BD and MN performed the experiments. IH, SO, BD and CP analysed the data. IH and CP wrote the paper. All authors read and approved the final manuscript.

## Supplementary Material

Additional file 1 **Diurnal pattern of phosphoenolpyruvate carboxylase (*Pepc*) promoter histone acetylation**. Histone acetylation in leaves 4 h after illumination (dark green lines), 16 h after illumination (light green lines) or re-etiolated leaves (orange lines), respectively. Values are H3K9, H3K14, H3K18, H3K23, H3K27, H4K5 and H4K16 histone acetylation levels at nine positions on the *Pepc *promoter. Data are standardized for acetylation levels on the *Actin*-1 promoter. For better orientation, the 1.0 level is emphasized by a black line. H3C = chromatin precipitated with an antibody to an invariant epitope on histone H3. Data points are based on four independent experiments. Vertical lines indicate standard errors. Click here for file

Additional file 2 **Comparison of nucleosome densities as determined by histone H3 and histone H4 occupancy**. Nucleosome density in leaves 4 h after illumination (dark green lines), 16 h after illumination (light green lines), or re-etiolated leaves (orange lines), respectively. H3C = chromatin precipitated with an antibody to an invariant epitope on histone H3; H4C = chromatin precipitated with an antibody to an invariant epitope on histone H4. Exemplarily, data for the phosphoenolpyruvate carboxylase (*Pepc*) core promoter and the *Actin*-1 promoter are given. Data points are based on four independent experiments. Vertical lines indicate standard errors. Click here for file

Additional file 3 **Circadian pattern of phosphoenolpyruvate carboxylase (*Pepc*) promoter histone acetylation**. Histone acetylation under constant illumination (free-running conditions). After a normal 16 h light period, illumination was extended for an additional 40 h without any dark period or temperature shift. Values are H3K9, H3K14, H3K18, H3K23, H3K27, H4K5 and H4K16 histone acetylation levels at nine positions on the *Pepc *promoter. The different lines represent acetylation levels at different time points during constant illumination. Green lines represent time points where high transcription levels were detected (solid line = 4 h after illumination (hai), dotted line = 24 hai, dashed line = 44 hai). Orange lines represent time points where low transcription levels were detected (solid line = 16 h hai, dotted line = 36 hai, dashed line = 56 hai). Data are standardized for acetylation levels on the *Actin*-1 promoter. For better orientation, the 1.0 level is emphasized by a black line. H3C = chromatin precipitated with an antibody to an invariant epitope on histone H3. Data points are based on four independent experiments. Vertical lines indicate standard errors. Click here for file

Additional file 4 Oligonucleotides and polymerase chain reaction conditions. Click here for file
