# Supplementary figures and images for: Core promoter acetylation is not required for high transcription from the phosphoenolpyruvate carboxylase promoter in maize
Source: Epigenetics Chromatin. 2009 Dec 2;2:17. doi: 10.1186/1756-8935-2-17 (PMC2793245; doi:10.1186/1756-8935-2-17)

## Additional file 1

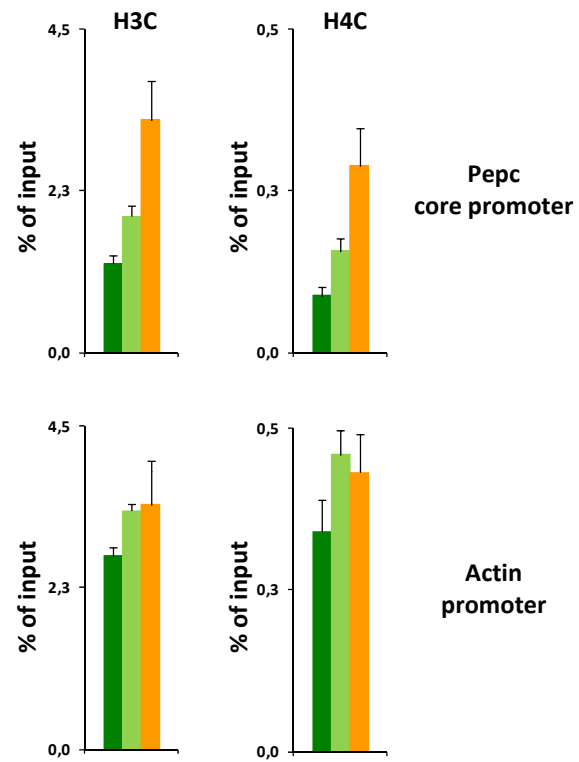

Supplement: Additional file 1 — Diurnal pattern of phosphoenolpyruvate carboxylase (Pepc) promoter histone acetylation. Histone acetylation in leaves 4 h after illumination (dark green lines), 16 h after illumination (light green lines) or re-etiolated leaves (orange lines), respectively. Values are H3K9, H3K14, H3K18, H3K23, H3K27, H4K5 and H4K16 histone acetylation levels at nine positions on the Pepc promoter. Data are standardized for acetylation levels on the Actin-1 promoter. For better orientation, the 1.0 level is emphasized by a black line. H3C = chromatin precipitated with an antibody to an invariant epitope on histone H3. Data points are based on four independent experiments. Vertical lines indicate standard errors. [file 1756-8935-2-17-S1.PDF]

## Additional file 2

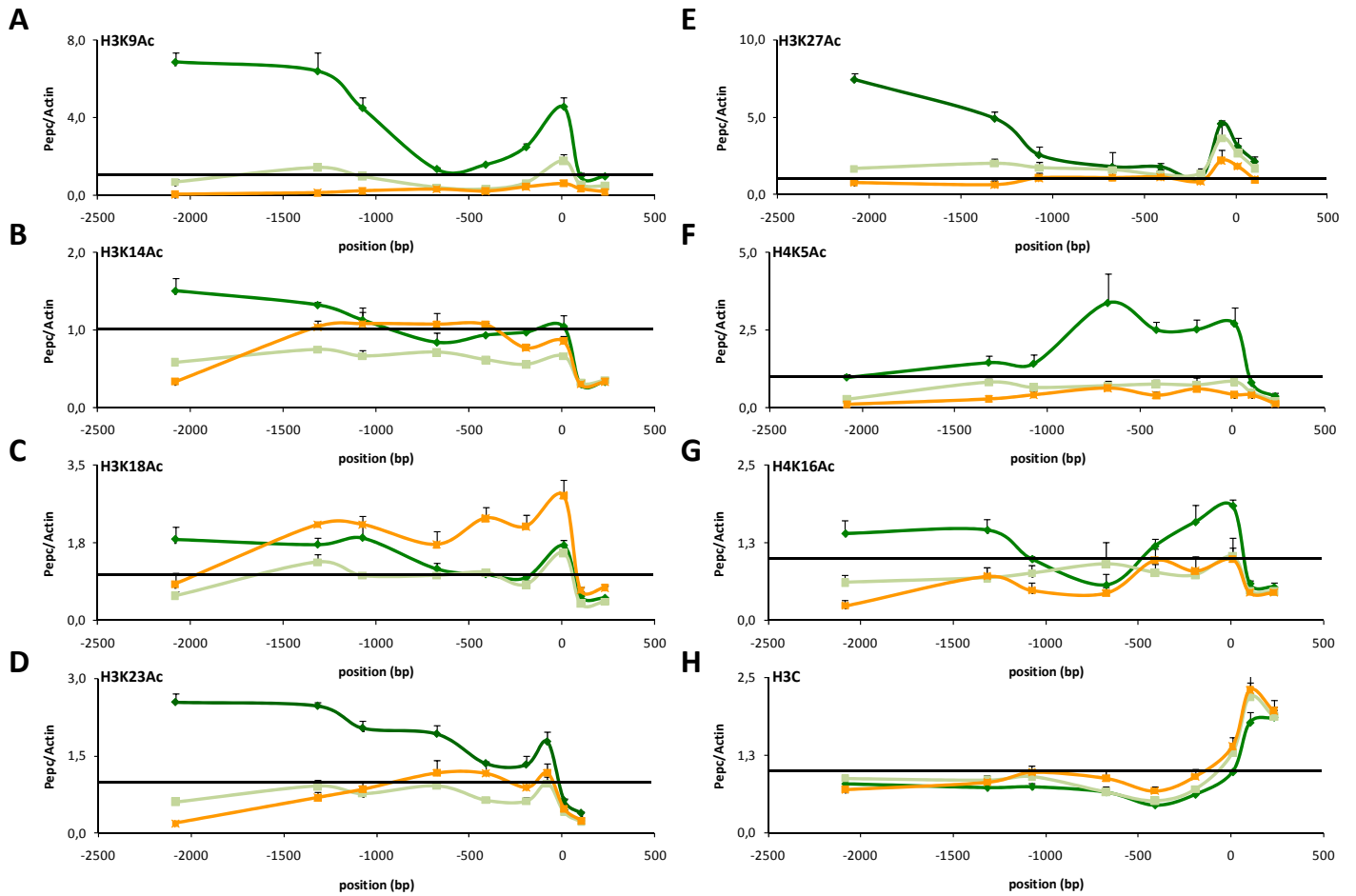

Supplement: Additional file 2 — Comparison of nucleosome densities as determined by histone H3 and histone H4 occupancy. Nucleosome density in leaves 4 h after illumination (dark green lines), 16 h after illumination (light green lines), or re-etiolated leaves (orange lines), respectively. H3C = chromatin precipitated with an antibody to an invariant epitope on histone H3; H4C = chromatin precipitated with an antibody to an invariant epitope on histone H4. Exemplarily, data for the phosphoenolpyruvate carboxylase (Pepc) core promoter and the Actin-1 promoter are given. Data points are based on four independent experiments. Vertical lines indicate standard errors. [file 1756-8935-2-17-S2.PDF]

## Additional file 3

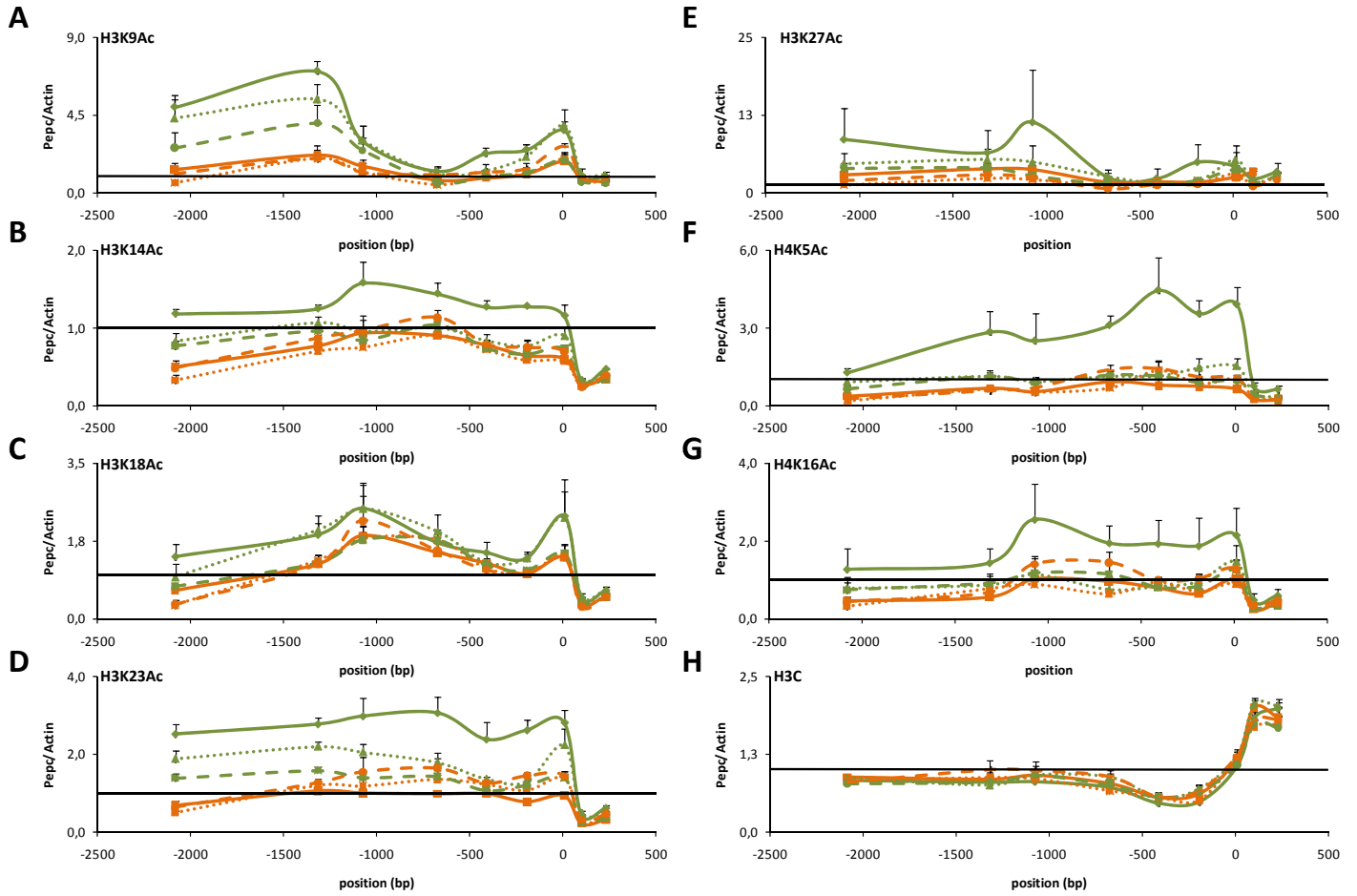

Supplement: Additional file 3 — Circadian pattern of phosphoenolpyruvate carboxylase (Pepc) promoter histone acetylation. Histone acetylation under constant illumination (free-running conditions). After a normal 16 h light period, illumination was extended for an additional 40 h without any dark period or temperature shift. Values are H3K9, H3K14, H3K18, H3K23, H3K27, H4K5 and H4K16 histone acetylation levels at nine positions on the Pepc promoter. The different lines represent acetylation levels at different time points during constant illumination. Green lines represent time points where high transcription levels were detected (solid line = 4 h after illumination (hai), dotted line = 24 hai, dashed line = 44 hai). Orange lines represent time points where low transcription levels were detected (solid line = 16 h hai, dotted line = 36 hai, dashed line = 56 hai). Data are standardized for acetylation levels on the Actin-1 promoter. For better orientation, the 1.0 level is emphasized by a black line. H3C = chromatin precipitated with an antibody to an invariant epitope on histone H3. Data points are based on four independent experiments. Vertical lines indicate standard errors. [file 1756-8935-2-17-S3.PDF]
