# Supplementary material for: Core promoter acetylation is not required for high transcription from the phosphoenolpyruvate carboxylase promoter in maize
Source: Epigenetics Chromatin. 2009 Dec 2;2:17. doi: 10.1186/1756-8935-2-17 (PMC2793245; doi:10.1186/1756-8935-2-17)
Supplement: Additional file 4 — Oligonucleotides and polymerase chain reaction conditions. [file 1756-8935-2-17-S4.PDF]

Additional file 4

Name/function	sequence (5' → 3')	Primer (nM)	$MgCl_2$ (mM)	Betain (M)	Product (bp)
-2100 f/r	GTCACAATTGAAGATTCGTGCAAGG / CAGTTTGAACTAAACGACTTCCAAC	200	3	0	224
-1300 f/r	GTACAAATGAGGTGCCGGATTGATG / CGGCCATGGCATGATACAATTCTCA	250	5	0	139
-1100 f/r	GGTGTTAGGACACGTGGTTAGC / CACTTGGCAGCGGTGAAGATAC	100	3	0	247
-700 f/r	TGGCACCCTTATCCCTACAATAGC / GTCTGTTTGCAGGATGTGGTTGAG	200	3	0	297
-400 f/r	CCCTCTCCACATCCTGCAAAGC / ATTCCGTTGGCTAATTGGGTAGCA	300	3	1	112
-200 f/r	CGATTGCCGCCAGCAGT / GAACCGGCTGTGGCTGAG	300	3	1	98
10 f/r	AACGACTCCCCATCCCTATTTGAAC / AGCAGGGAAGCGAGACGGTTG	200	3	0.5	110
100 f/r	CCCTGCTTAGCTTCCCGCCG / TGACGGAGCTGCGCGTCGATG	200	3	0	87
200 f/r	AAGGTCTCCGAGGACGACAAG / ACCGGTCGAAGAAGAAATGGG	200	3	0	142
Actin-1 f/r	TTTAAGGCTGCTGTACTGCTGTAGA / CACTTTCTGCTCATGGTTTAAGG	200	3	0	119
Zein f/r	ACGTTGTCCGGTAGAGAGGGTAG / CGAACTAGCAAGCTGGAGCTCTG	400	4	0.75	105
Pepc mRNA f/r	AGAACTCAAGCCCTTTGGGAAGC / GTCGGCGAACTCCTTGGACAGC	300	3	0	241
Pepc hnRNA f/r (coding system)	GTATGCTGCCATTGCCCATTGC / GTCTCCGGTGTAGCCTGATAGTGA	300	5	1	110
Actin-1 mRNA f/r	CCTATCGTATGTGACAATGGCACT / GCCTCATCACCTACGTAGGCAT	200	3	0	119
